# Correlation of fractional exhaled nitric oxide levels with inflammation and lung function in patients with chronic obstructive pulmonary disease at different stages: A retrospective study

**DOI:** 10.1097/MD.0000000000046615

**Published:** 2026-01-02

**Authors:** Chunfeng Sheng, Yanyan Zhao, Peng Wu, Lingxia Wang, Yongqin Yan, Qingfeng Jin

**Affiliations:** a Department of Respiratory and Critical Medicine, Songjiang Hospital Affiliated to Shanghai Jiao Tong University School of Medicine, Shanghai, China; b Department of General Practice, Yongfeng Community Health Service Center of Songjiang, Shanghai, China.

**Keywords:** airway inflammation, COPD, FeNO, inflammatory markers, lung function

## Abstract

This study aims to evaluate the levels of fractional exhaled nitric oxide (FeNO) in patients with chronic obstructive pulmonary disease (COPD) at different stages, analyze its correlation with inflammatory markers and lung function indices, and explore the feasibility of FeNO as a potential biomarker for airway inflammation in COPD patients. A retrospective analysis was conducted on 146 COPD patients covering the period from January 2021 to June 2023. Among them, 87 patients in the acute exacerbation phase were designated as group B, 59 patients in the stable phase as group C, and 30 healthy controls were selected as group A. Lung function was assessed by measuring predicted forced expiratory volume in the first second (FEV1)%pred and the FEV1/forced vital capacity ratio, while FeNO levels and various inflammatory markers (C-reactive protein, d-dimer, interleukin [IL]-2, IL-6) were also measured. Pearson correlation analysis was conducted to explore the correlation of FeNO with lung function and inflammatory markers. Patients with COPD at the acute exacerbation phase had significantly higher FeNO levels than the other 2 groups (*P* < .001), and these levels were negatively correlated with FEV1%pred and the FEV1/forced vital capacity ratio. FeNO levels were positively correlated with inflammatory markers C-reactive protein, d-dimer, and IL-6 (*P* < .01). Eosinophil counts in peripheral blood and induced sputum showed a significant positive correlation with FeNO levels. FeNO levels significantly increased during acute exacerbation phase of COPD and were associated with declines in lung function and elevated levels of inflammatory markers. FeNO could serve as a potential monitoring indicator for airway inflammation in COPD, aiding in the assessment and management of the condition.

## 1. Introduction

Chronic obstructive pulmonary disease (COPD) exhibits a high prevalence in clinical settings and is categorized as a respiratory system disorder. According to reports, as of 2019, over 200 million people worldwide suffer from COPD, with more than 3 million fatalities attributed to the disease, severely impacting global public health.^[1]^ The pathogenesis of COPD is intricate, involving airway inflammation, airway remodeling, and alterations in lung structure.^[2,3]^ Research^[4,5]^ indicates that the progression of COPD is closely associated with various inflammatory factors and biomarkers. Among these, fractional exhaled nitric oxide (FeNO) is gaining increasing attention as a biomarker for airway inflammation. FeNO is a gas produced by nitric oxide synthase in the respiratory tract, reflecting the level of nitric oxide generation within the airways.^[6]^ Research indicates that elevated FeNO levels are generally associated with exacerbation of airway inflammation.^[7]^ Therefore, FeNO is extensively utilized as a tool for assessing airway inflammation and disease activity. The variation in FeNO levels in patients with COPD at different stages provides crucial insights into the severity of airway inflammation. Research^[8]^ indicates that the FeNO indicator can be utilized to assess inflammation in the small airways of bronchial asthma, serving as a criterion for functional evaluation and as an adjunctive marker for treatment. In recent years, research has focused on the relationship between FeNO and small airway changes in COPD, yet the correlation between FeNO and COPD remains inconclusive. Therefore, this study aims to address the following questions: whether FeNO levels significantly differ across disease stages in patients with COPD, and whether FeNO levels are correlated with inflammatory markers and lung function decline.

## 2. Methods

### 2.1. General data

**Figure 1.**
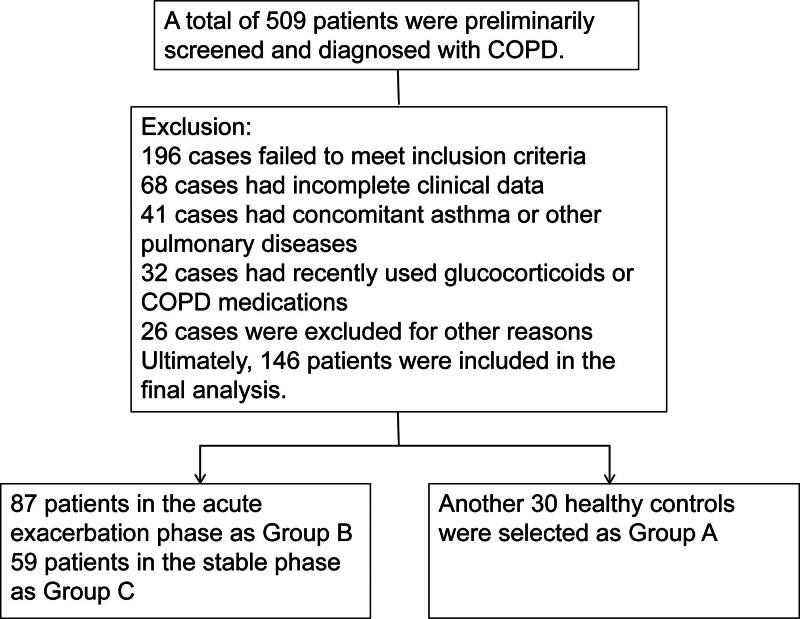
Flowchart of patient selection for the study.

This single-center retrospective cohort study was approved by Songjiang Hospital Affiliated to Shanghai Jiao Tong University School of Medicine Ethics Committee. Given the retrospective nature of the study, the requirement for informed consent was waived by the Ethics Committee. This study fully complied with the ethical principles of medical research as outlined in the Declaration of Helsinki (World Medical Association). The study period spanned from January 2021 to June 2023, including 146 cases of COPD for analysis. Among them, 87 patients in the acute phase of COPD were designated as group B, comprising 50 males and 37 females, aged 47 to 75 years, with an average age of (65.17 ± 4.55) years. Thirty-three patients had a history of smoking. Another 59 patients in the stable phase of COPD were assigned to group C, including 35 males and 24 females, aged 44 to 75 years, with an average age of (64.36 ± 4.79) years. Twenty patients had a history of smoking. Additionally, 30 individuals undergoing physical examinations at our hospital during the same period, with no history of pulmonary diseases and a ratio of forced expiratory volume in the first second and forced vital capacity (FEV1/FVC) of ≥83%, were selected as group A, consisting of 18 males and 12 females, aged 43 to 75 years, with an average age of (63.52 ± 3.98) years. Ten patients had a history of smoking. There were no significant differences in age, sex, and other demographic data among the 3 groups (*P* > .05). The clinical data included in this study were verified for completeness, and no missing values were detected. Therefore, all statistical analyses were performed based on complete-case analysis. The case inclusion process is shown in Figure 1.

This was a single-center retrospective study, and no prior sample size estimation was performed. post hoc power analysis was conducted using G*Power 3.1 software (α = 0.05). The power for detecting differences in FeNO among the 3 groups was approximately 0.90, while the power for testing the correlation between FeNO and FEV1%pred exceeded 0.99, indicating that the current sample size was sufficient to ensure statistical detection power of the main effect.

### 2.2. Inclusion and exclusion criteria

#### 2.2.1. Inclusion criteria

Patients diagnosed as COPD according to the COPD diagnostic criteria outlined in the Guidelines for the Diagnosis and Treatment of Chronic Obstructive Pulmonary Disease (2013 edition)^[9]^ prepared by the Chronic Obstructive Pulmonary Disease Group of the Respiratory Disease Branch of the Chinese Medical Association, with COPD categorized into acute and stable phases, and FEV1/FVC < 70%; No history of steroid use within 30 days prior to hospital admission; Absence of autoimmune diseases; No severe hepatic or renal dysfunction; Age between 18 and 75 years.

#### 2.2.2. Exclusion criteria

Patients with concurrent asthma, allergic rhinitis, pleural effusion, organic lung diseases, severe pulmonary infections, malignant pulmonary tumors or space-occupying lesions, or chest trauma deformities; those whose FeNO levels were influenced by various confidential factors; Those with a history of hormone or COPD medication treatment within 1 month of enrollment; Individuals under 18 or over 75 years of age; Those with incomplete clinical data.

### 2.3. Study methods

#### 2.3.1. Lung function tests

Pulmonary function tests included the percentage of predicted forced expiratory volume in the first second (FEV1%pred) and the ratio of FEV1/FVC. These parameters were measured using a Master Screen spirometer (Jaeger, Germany).

#### 2.3.2. Determination of inflammatory markers and serum FeNO

Following the standardized measurement procedure recommended by the American Thoracic Society/European Respiratory Society in the “FeNO Testing Guidelines,” the operation was conducted as follows: After the subject inhaled through a specialized filter to eliminate exogenous nitric oxide gas, they exhaled at a flow rate of 50 mL/s after reaching their maximum lung capacity. The exhalation pressure was maintained at 10 to 20 cm H_2_O, and the nitric oxide analyzer was used to automatically calculate the results, taking the average of 3 measurements. FeNO (ppb) = detected result × 10^–9^ mol/L. The detection of inflammatory markers such as C-reactive protein (CRP), d-dimer, interleukin (IL)-2, and IL-6 was performed using the enzyme-linked immunosorbent assay method, with the test kits supplied by Jiangsu Baolai Biotechnology Co., Ltd. The entire testing process adhered strictly to the kit’s operational instructions.

#### 2.3.3. Detection of peripheral blood eosinophil (Eos) and induced sputum Eos levels

A venous blood sample of 4 mL was collected from the elbow for examination. The sample was centrifuged under the following parameters: a speed of 3000 rpm for 3 minutes. The Eos expression level was then analyzed using an automated blood cell analyzer (Beckman Coulter, Inc.).

#### 2.3.4. Treatment

The clinical data should include the treatment plan for patients in the acute phase. Group C patients during the acute exacerbation received standardized treatment, including inhaled glucocorticoids, bronchodilators, antibiotics, and oxygen therapy. Vital signs were regularly monitored during treatment, and indicators such as Eos, neutrophils (N), FeNO, and FEV1 were measured again within 1 week after treatment. During the 12-month follow-up period, the number of acute exacerbations, number of hospitalizations, and related complications were recorded.

### 2.4. Statistical analysis

Data analysis was performed using IBM SPSS Statistics 25.0 software (IBM Corp., Armonk). Continuous variables were expressed as mean ± standard deviation, and inter-group comparisons were conducted using one-way analysis of variance or independent samples *t* test. Non-continuous variables between groups were assessed with the chi-square test. The correlation of FeNO levels with lung function and inflammatory markers was evaluated through Pearson correlation analysis. The sensitivity and specificity of FeNO in diagnosing severe COPD during the stable phase were assessed using ROC curve analysis. Statistical significance was set at *P* < .05.

## 3. Results

### 3.1. Comparison of lung function

**Figure 2.**
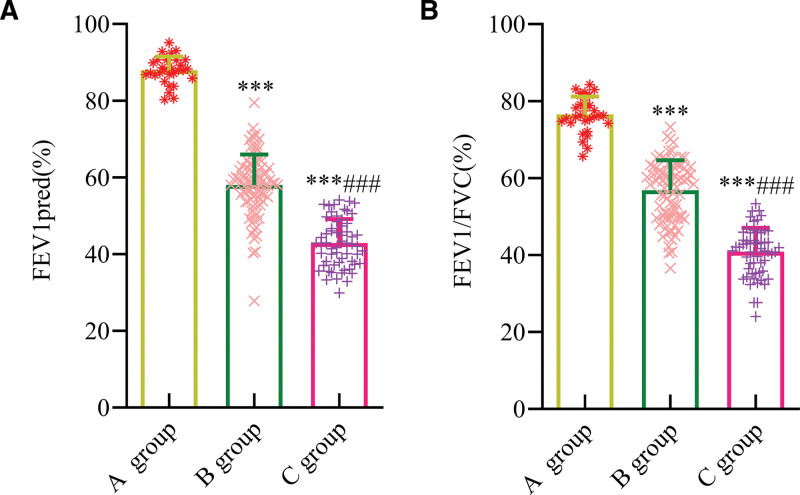
Comparison of lung function levels in patients with COPD at different stages. (A) FEV1pred; (B) FEV1/FVC. Compared with group A, ^***^P < .001; compared with group B, ^###^*P* < .001. FEV1pred = predicted forced expiratory volume in the first second, FEV1/FVC = the ratio of forced expiratory volume in the first second and forced vital capacity, Group A = healthy controls, Group B = patients in the acute exacerbation phase, Group C = patients in the stable phase.

The differences in FEV1pred and FEV1/FVC were statistically significant among the 3 groups (*P* < .001). The FEV1pred and FEV1/FVC values in group B were significantly lower than those in group A (*P* < .001), while these indicators in group C were markedly lower than in both group A and group B (*P* < .001) (Fig. 2).

### 3.2. Comparison of FeNO levels

**Figure 3.**
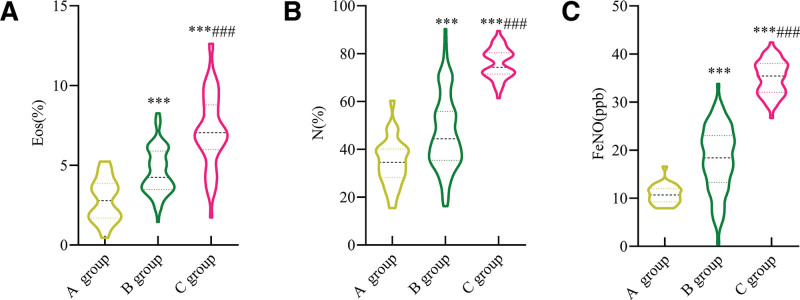
Comparison of FeNO levels in patients with COPD at different stages. (A) Eos; (B) N; (C) FeNO. Compared with group A, ^***^P < .001; compared with group B, ^###^*P* < .001. Eos = eosinophils, N = neutrophils, FeNO = fractional exhaled nitric oxide’ Group A = healthy controls, Group B = patients in the acute exacerbation phase, Group C = patients in the stable phase.

The differences in Eos, N, and FeNO levels were statistically significant among the 3 groups before treatment (*P* < .001). The levels of Eos, N, and FeNO in group C were significantly higher than those in groups B and A (*P* < .001), while these indicators in group B were markedly higher than in group A (*P* < .001) (Fig. 3).

### 3.3. Comparison of CRP, d-dimer, IL-2, and IL-6 levels

**Figure 4.**
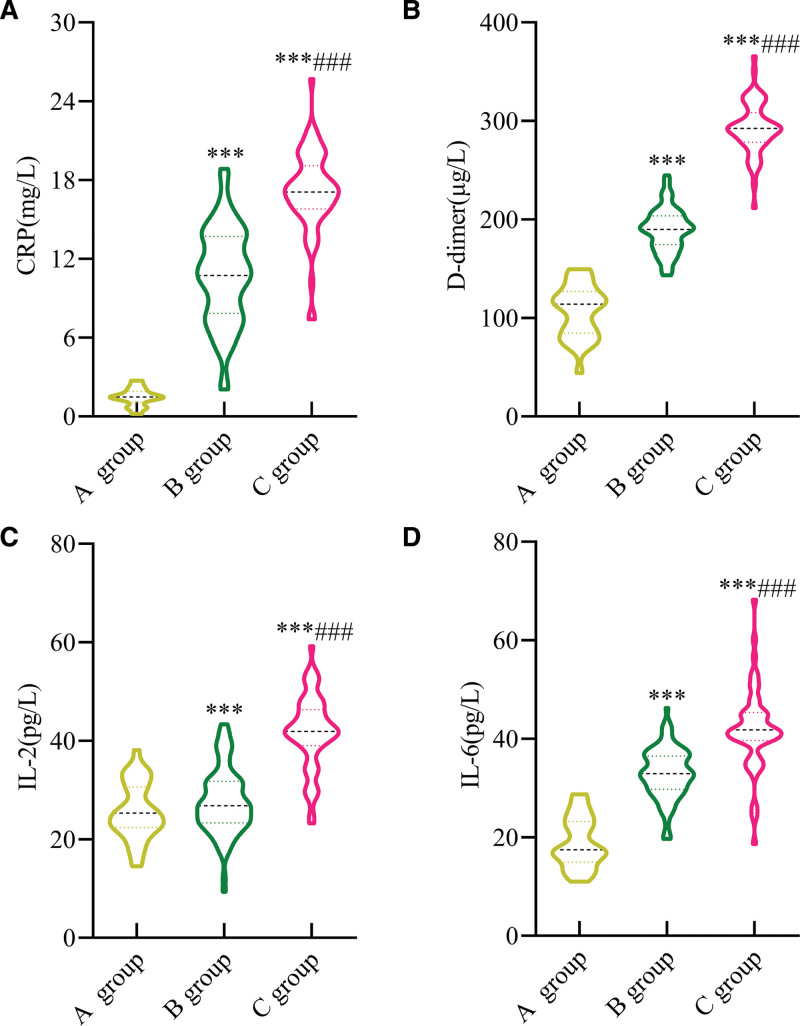
Comparison of CRP, d-dimer, IL-2, and IL-6 levels. (A) CRP; (B) d-dimer; (C) IL-2; (D) IL-6. Compared with group A, ^***^P < .001; compared with group B, ^###^*P* < .001. CRP = C-reactive protein, Group A = healthy controls, Group B = patients in the acute exacerbation phase, Group C = patients in the stable phase, IL-2 = interleukin-2, IL-6 = interleukin-6.

The differences in CRP, d-dimer, IL-2, and IL-6 levels were statistically significant among the 3 groups before treatment (*P* < .001). The levels of CRP, d-dimer, IL-2, and IL-6 in group C were significantly higher than those in groups B and A (*P* < .001), while these indicators in group B were notably higher than those in group A (*P* < .001) (Fig. 4).

### 3.4. Comparison of peripheral blood Eos and induced sputum Eos levels

Compared to group A, the peripheral blood Eos and induced sputum Eos levels of groups B and C were significantly elevated (*P* < .001), while there was no significant difference between groups B and C (*P* > .05), as shown in Table 1.

**Table 1:** Comparison of peripheral blood eosinophil and induced sputum eosinophil levels.

Group	Peripheral blood eosinophil (%)	Induced sputum eosinophil (%)
Group A	2.95 ± 0.72 (95% CI: 2.72–3.18)	2.66 ± 0.75 (95% CI: 2.41–2.91)
Group B	7.17 ± 2.66 (95% CI: 6.58–7.76)***	6.31 ± 2.92 (95% CI: 5.67–6.95)***
Group C	7.82 ± 2.53 (95% CI: 7.24–8.40)***,###	6.46 ± 2.33 (95% CI: 5.94–6.98)***,###
*F*	44.775	27.909
*P*	<.001	<.001

CI = confidence interval.

*** Compared with group A, *P* < .001.

### Compared with group B, *P* < .001.

### 3.5. Comparison of Eos, N, FeNO, and FEV1 levels before and after treatment during acute exacerbation phase of COPD

At 1 week after treatment in group C, the levels of Eos, N, FeNO, and FEV1 were significantly lower than before treatment (*P* < .001) and higher than those in group A (*P* < .001). However, there was no difference in these indicators between groups C and B at 1 week after treatment (*P* > .05), as shown in Table 2.

**Table 2:** Comparison of Eos, N, FeNO, and FEV1 levels before and after treatment during acute exacerbation phase of COPD.

Group	Eos (%)	N (%)	FeNO (ppb)	FEV1 (%)
Group A	2.88 ± 1.23 (95% CI: 2.54–3.22)	34.69 ± 9.88 (95% CI: 32.06–37.32)	10.82 ± 1.93 (95% CI: 10.31–11.33)	87.96 ± 3.57 (95% CI: 86.74–89.18)
Group B	4.62 ± 1.47 (95% CI: 4.23–5.01)***	46.95 ± 15.72 (95% CI: 43.16–50.74)***	18.23 ± 6.78 (95% CI: 16.60–19.86)***	58.05 ± 7.94 (95% CI: 56.05–60.05)
Group C				
Before treatment	7.22 ± 2.32 (95% CI: 6.63–7.81)***,###	75.91 ± 6.33 (95% CI: 74.38–77.44)***,###	35.08 ± 3.56 (95% CI: 34.25–35.91)***,###	42.88 ± 6.29 (95% CI: 41.36–44.40)***,###
After treatment	4.83 ± 1.66 (95% CI: 4.41–5.25)***,&&&	45.09 ± 10.24 (95% CI: 42.71–47.47)***,&&&	19.07 ± 8.47 (95% CI: 17.11–21.03)***,&&&	57.14 ± 8.31 (95% CI: 55.22–59.06)***,&&&

CI = confidence interval, COPD = chronic obstructive pulmonary disease, CRP = C-reactive protein, Eos = eosinophils, FeNO = fractional exhaled nitric oxide, FEV1 = predicted forced expiratory volume in the first second, N = neutrophils.

*** Compared with group A, *P* < .001.

### Compared with group B, *P* < .001.

&&& Compared with group C, *P* < .001.

### 3.6. Comparison of inflammatory factor levels before and after treatment during acute exacerbation phase of COPD

At 1 week after treatment in group C, the levels of inflammatory markers were significantly lower than before treatment (*P* < .001) and higher than those in group A (*P* < .001). However, when compared to group B, the markers in group C showed no significant difference at 1 week after treatment (*P* > .05), as shown in Table 3.

**Table 3:** Comparison of inflammatory factor levels before and after treatment during acute exacerbation phase of COPD.

Group	CRP	d-Dimer	IL-2	IL-6
Group A	1.48 ± 0.66 (95% CI 1.31–1.65)	106.88 ± 26.78 (95% CI 100–114)	25.66 ± 5.82 (95% CI 24.2–27.1)	41.88 ± 7.38 (95% CI 40.0–43.8)
Group B	10.72 ± 3.67 (95% CI 9.9–11.5)***	189.22 ± 22.08 (95% CI 184–194)***	27.63 ± 6.63 (95% CI 26.2–29.1)***	32.93 ± 5.12 (95% CI 31.7–34.2)***
Group C				
Before treatment	17.09 ± 3.17 (95% CI 16.3–17.9)***,###	292.37 ± 26.72 (95% CI 287–298)***,###	41.88.±7.38 (95% CI 40.1–43.7)***,###	41.82 ± 8.12 (95% CI 40.0–43.6)***,###
After treatment	11.02 ± 3.93 (95% CI 10.2–11.8)***,&&&	193.45 ± 27.81 (95% CI 188–199)***,&&&	28.55 ± 8.27 (95% CI 26.9–30.2)***,&&&	31.274 ± 7.71 (95% CI 29.7–32.8)***,&&&

CI = confidence interval, COPD = chronic obstructive pulmonary disease, CRP = C-reactive protein, IL = interleukin.

*** Compared with group A, *P* < .001.

### Compared with group B, *P* < .001.

&&& Compared with group C, *P* < .001.

### 3.7. Correlation of serum FeNO levels with lung function and inflammatory markers

**Figure 5.**
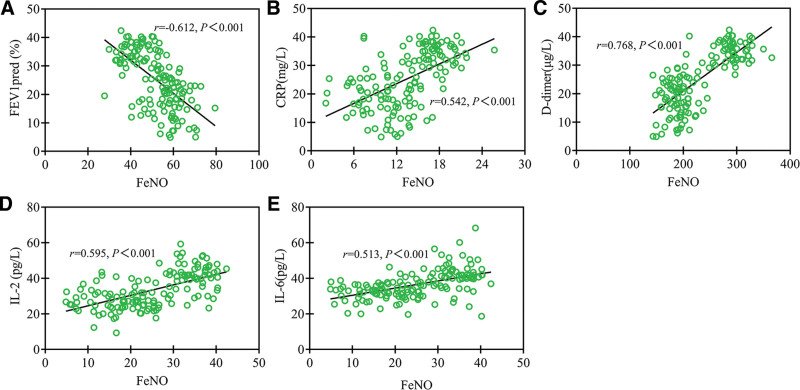
Correlation of serum FeNO levels with lung function and inflammatory markers. (A) FEV1pred; (B) CRP; (C) d-dimer; (D) IL-2; (E) IL-6. CRP = C-reactive protein, FeNO = fractional exhaled nitric oxide, FEV1pred = predicted forced expiratory volume in the first second, IL-2 = interleukin-2.

The Pearson correlation analysis revealed a significant negative correlation between serum FeNO levels and the lung function index FEV1pred in COPD patients (*P* < .001), as well as a significant positive correlation with serum pro-inflammatory cytokines (*P* < .001). These findings suggest that elevated FeNO levels reflect not only worsening airway obstruction but also heightened systemic inflammatory responses (Fig. 5).

### 3.8. Comparison of number of acute exacerbation in patients with different FeNO levels

All patients completed the 12-month follow-up without any loss to follow-up. After 12 months of follow-up, the number of acute exacerbations in the high FeNO group was significantly higher than in the low FeNO group (*P* < .05). This finding suggests that patients with elevated baseline FeNO levels are more susceptible to future exacerbations, which may be associated with persistent airway inflammation during clinically stable periods, as shown in Table 4.

**Table 4:** Comparison of number of acute exacerbation in patients with different FeNO levels.

Group	0	1	≥2 times	Average number of times
Low FeNO group (n = 79)	15	61	3	0.85 ± 0.51
High FeNO group (n = 67)	0	50	17	1.31 ± 0.68
*Z*				4.663
*P*				<.001

Eos = eosinophil, FeNO = fractional exhaled nitric oxide.

### 3.9. Diagnostic value of FeNO in severe COPD during the stable phase

**Figure 6.**
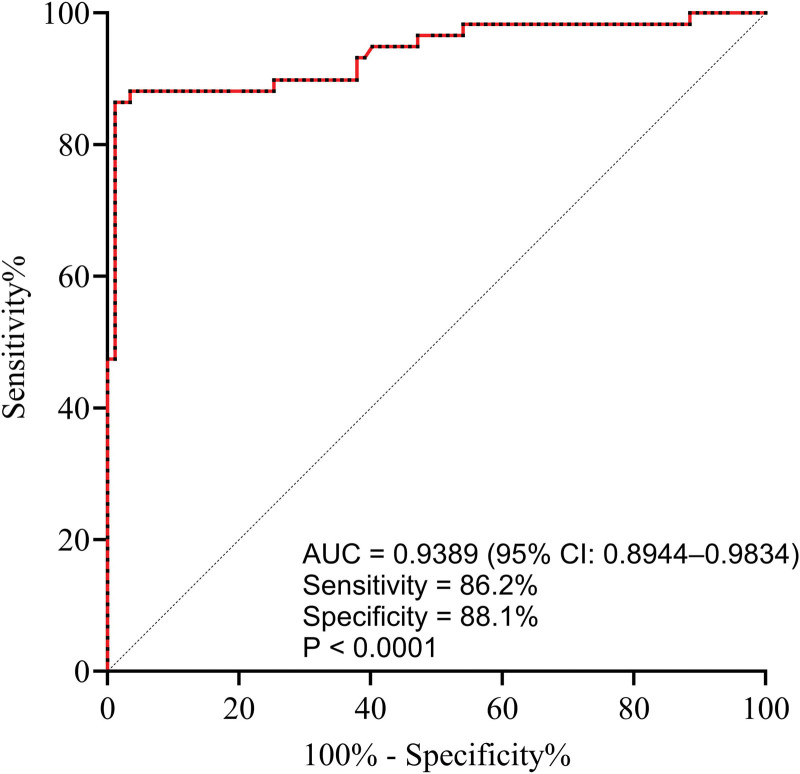
Diagnostic value of FeNO in severe COPD during the stable phase.

ROC curve analysis revealed that FeNO had significant diagnostic value in identifying severe COPD during the stable phase (AUC = 0.939, *P* < .001) (Fig. 6).

## 4. Discussion

This study explored the role of FeNO in COPD patients at different stages and analyzed its correlation with lung function, inflammatory factors, and Eos levels. The study suggests that patients with acute exacerbations of COPD exhibit the poorest lung function and the highest levels of FeNO, indicating that FeNO may potentially serve as a biomarker for airway inflammation. The elevation of FeNO levels reflects an increase in airway inflammation, thereby contributing to the decline in lung function.

Inflammatory factors play a crucial role in the onset and progression of COPD.^[10,11]^ CRP, d-dimer, IL-2, and IL-6 serve as inflammatory markers in COPD; variations in their levels can reflect the severity of airway inflammation, the status of systemic inflammation, and the risk of acute exacerbations. Research indicates that elevated CRP levels correlate with exacerbations in COPD, with high levels typically indicating the presence of systemic inflammation.^[12,13]^ d-Dimer is a marker of thrombosis, with elevated levels associated with inflammation and thrombus formation in COPD.^[14,15]^ IL-2 and IL-6 are involved in the modulation of airway inflammation and immune response, with elevated IL-6 levels particularly correlating with the severity of airway inflammation.^[16,17]^ Our results indicated that patients with COPD in exacerbation phase exhibited significantly higher levels of CRP, d-dimer, IL-2, and IL-6 prior to treatment compared to those in stable phase, further confirming the role of these inflammatory factors as indicators of disease severity in COPD. Consistent with previous studies, the positive correlation between FeNO levels and these inflammatory factors suggests that FeNO can serve as an effective marker for reflecting airway inflammation.

The role of Eos in COPD has also garnered significant attention. An elevation in peripheral blood Eos and induced sputum Eos levels is typically associated with exacerbation of airway inflammation.^[18,19]^ We found that in patients with COPD in exacerbation phase, serum Eos levels were significantly higher compared to those in stable phase, indicating the degree of airway inflammation in COPD patients, and that Eos levels markedly increased during the acute exacerbation phase, which was consistent with elevated FeNO levels.^[20]^

FeNO levels exhibit significant variation at different stages of COPD. During the stable phase, FeNO levels are typically low, whereas during the acute exacerbation phase, FeNO levels rise significantly.^[21,22]^ This is due to acute exacerbation accompanied by intensified airway inflammation, leading to increased production of nitric oxide. Our research revealed that, at 1 week after treatment, patients in the acute exacerbation phase showed a significant reduction in FeNO levels. This indicates that, although the treatment effectively lowers FeNO levels during the acute exacerbation phase, these levels remain elevated compared to stable-phase patients, suggesting that the inflammatory state from the acute exacerbation has not fully resolved after treatment. Moreover, the number of acute exacerbations was significantly higher in the high FeNO group compared to the low FeNO group, further indicating a correlation between FeNO levels and the frequency of acute exacerbations. This finding is consistent with previous research, as elevated FeNO levels generally indicate the persistent presence of airway inflammation and may predict the risk of acute exacerbations.

The ROC curve analysis indicates that FeNO holds significant diagnostic value in identifying severe COPD during stable phase. This demonstrates the importance of FeNO in COPD diagnosis, effectively distinguishing patients with severe COPD during stable phase and providing a robust auxiliary diagnostic tool for clinical use.

Although this study reveals significant associations between FeNO, inflammatory factors, and lung function indicators in COPD patients, there are still shortcomings. This study is a retrospective analysis limited by the sample size, which may affect the generalizability and broad applicability of the results. Large-scale multicenter studies will be conducted to better validate and extend the conclusions of this research. Moreover, this study is a single-center retrospective analysis, which may have introduced selection bias. Due to incomplete documentation of key confounding factors such as smoking status, environmental exposure, or prior treatment regimens in some cases, these variables could not be adjusted for in the statistical analysis. This may have impacted the accuracy of the association between FeNO levels and inflammatory markers to some extent. Future prospective studies should incorporate these variables to enhance the reliability of the findings. Furthermore, the expression and predictive capabilities of FeNO as a biomarker in different COPD subtypes require further in-depth research. Finally, this study did not conduct long-term follow-up on COPD patients, making it impossible to assess the dynamic changes in FeNO levels in long-term disease management and their ability to predict disease progression. Therefore, in the future research, these limitations should be taken into account to gain a more comprehensive understanding of the clinical significance of FeNO in COPD.

As a single-center retrospective study, the external validity of its findings may be limited. Although the study population to some extent represents the characteristics of COPD patients in this region, regional variations and differences in clinical practice may influence FeNO levels and inflammatory responses. Therefore, future multicenter, large-scale studies are needed to validate these findings. Concurrently, prospective studies should be designed to further confirm the value of FeNO as a predictive biomarker for COPD exacerbations and disease progression.

In conclusion, FeNO levels were significantly correlated with lung function, inflammatory markers, and the frequency of acute exacerbations, making it an essential tool for assessing airway inflammation, predicting acute exacerbations, and diagnosing COPD. Future research should further explore the role of FeNO in COPD treatment and evaluate its potential in personalized therapeutic strategies.

## Author contributions

**Conceptualization:** Chunfeng Sheng, Yanyan Zhao, Yongqin Yan.

**Data curation:** Yanyan Zhao, Yongqin Yan.

**Formal analysis:** Yanyan Zhao, Lingxia Wang.

**Investigation:** Lingxia Wang.

**Methodology:** Chunfeng Sheng, Peng Wu, Lingxia Wang, Qingfeng Jin.

**Project administration:** Chunfeng Sheng, Peng Wu, Qingfeng Jin.

**Validation:** Peng Wu.

**Writing – original draft:** Chunfeng Sheng, Qingfeng Jin.

**Writing – review & editing:** Yongqin Yan, Qingfeng Jin.
